# Identification of Functionally Important Residues of the Rat P2X4 Receptor by Alanine Scanning Mutagenesis of the Dorsal Fin and Left Flipper Domains

**DOI:** 10.1371/journal.pone.0112902

**Published:** 2014-11-14

**Authors:** Vendula Tvrdonova, Milos B. Rokic, Stanko S. Stojilkovic, Hana Zemkova

**Affiliations:** 1 Department of Cellular and Molecular Neuroendocrinology, Institute of Physiology Academy of Sciences of the Czech Republic, Prague, Czech Republic; 2 Department of Physiology of Animals, Faculty of Science, Charles University, Prague, Czech Republic; 3 Section on Cellular Signaling, Program in Developmental Neuroscience, National Institute of Child Health and Human Development, National Institutes of Health, Bethesda, Maryland, United States of America; University of Exeter, United Kingdom

## Abstract

Crystallization of the zebrafish P2X4 receptor in both open and closed states revealed conformational differences in the ectodomain structures, including the dorsal fin and left flipper domains. Here, we focused on the role of these domains in receptor activation, responsiveness to orthosteric ATP analogue agonists, and desensitization. Alanine scanning mutagenesis of the R203-L214 (dorsal fin) and the D280-N293 (left flipper) sequences of the rat P2X4 receptor showed that ATP potency/efficacy was reduced in 15 out of 26 alanine mutants. The R203A, N204A, and N293A mutants were essentially non-functional, but receptor function was restored by ivermectin, an allosteric modulator. The I205A, T210A, L214A, P290A, G291A, and Y292A mutants exhibited significant changes in the responsiveness to orthosteric analog agonists 2-(methylthio)adenosine 5′-triphosphate, adenosine 5′-(γ-thio)triphosphate, 2′(3′-O-(4-benzoylbenzoyl)adenosine 5′-triphosphate, and α,β-methyleneadenosine 5′-triphosphate. In contrast, the responsiveness of L206A, N208A, D280A, T281A, R282A, and H286A mutants to analog agonists was comparable to that of the wild type receptor. Among these mutants, D280A, T281A, R282A, H286A, G291A, and Y292A also exhibited increased time-constant of the desensitizing current response. These experiments, together with homology modeling, indicate that residues located in the upper part of the dorsal fin and left flipper domains, relative to distance from the channel pore, contribute to the organization of the ATP binding pocket and to the initiation of signal transmission towards residues in the lower part of both domains. The R203 and N204 residues, deeply buried in the protein, may integrate the output signal from these two domains towards the gate. In addition, the left flipper residues predominantly account for the control of transition of channels from an open to a desensitized state.

## Introduction

The purinergic P2X receptors (P2XRs) are ATP-gated ion channels that are permeable to Na^+^, K^+^, Ca^2+^, and small organic cations. Seven subunits of P2XRs have been identified in mammals [1], and functional receptors are composed of three homologous or heterologous subunits [2]. Each subunit consists of a large, glycosylated, and cystine-rich extracellular domain that contributes to the formation of the intersubunit ATP binding sites, two transmembrane domains that form the pore of the channel, and intracellular N- and C- termini that contribute to gating specificity [3]. Previous studies using single-point mutagenesis have identified most of conserved amino acid residues involved in ATP binding and have shown that ATP binding occurs at the interface between adjacent receptor subunits, assuming that ATP stabilizes the P2X trimer [4]–[10]. In contrast to the large number of studies using the native ligand, ATP, there are very few studies providing structural information derived from the use of orthosteric ATP analog agonists. Understanding receptor interactions with these analog agonists may provide significant insights aiding the design of drugs that compete with the native ligand.

The recent crystallization of the zebrafish P2X4R receptor (zfP2X4R) in the absence (closed state; PDB entry codes: 3H9V and 4DW0) and presence (open state; 4DW1) of ATP has confirmed the predicted topology and locations of the ATP binding sites in P2XRs. The authors suggest that the architecture of the P2XRs resembles a dolphin, with a rigid central extracellular body domain, a flexible head, a left flipper (LF), a right flipper, and a dorsal fin (DF). The crystal structure of zfP2XR in the apo-closed state and the ATP-bound open state has also provided structural insights into the mechanisms of ATP binding, the opening of ion channel pore, and a series of conformational changes associated with channel gating [11], [12]. These insights have enabled a better understanding of precrystallization studies focused on the structural-functional characterization of P2XR transmembrane domains [13]–[21] and facilitated further studies focused on extracellular vestibule function [22]–[24] and molecular dynamics to model conformation transitions [25].

**Figure 1:**
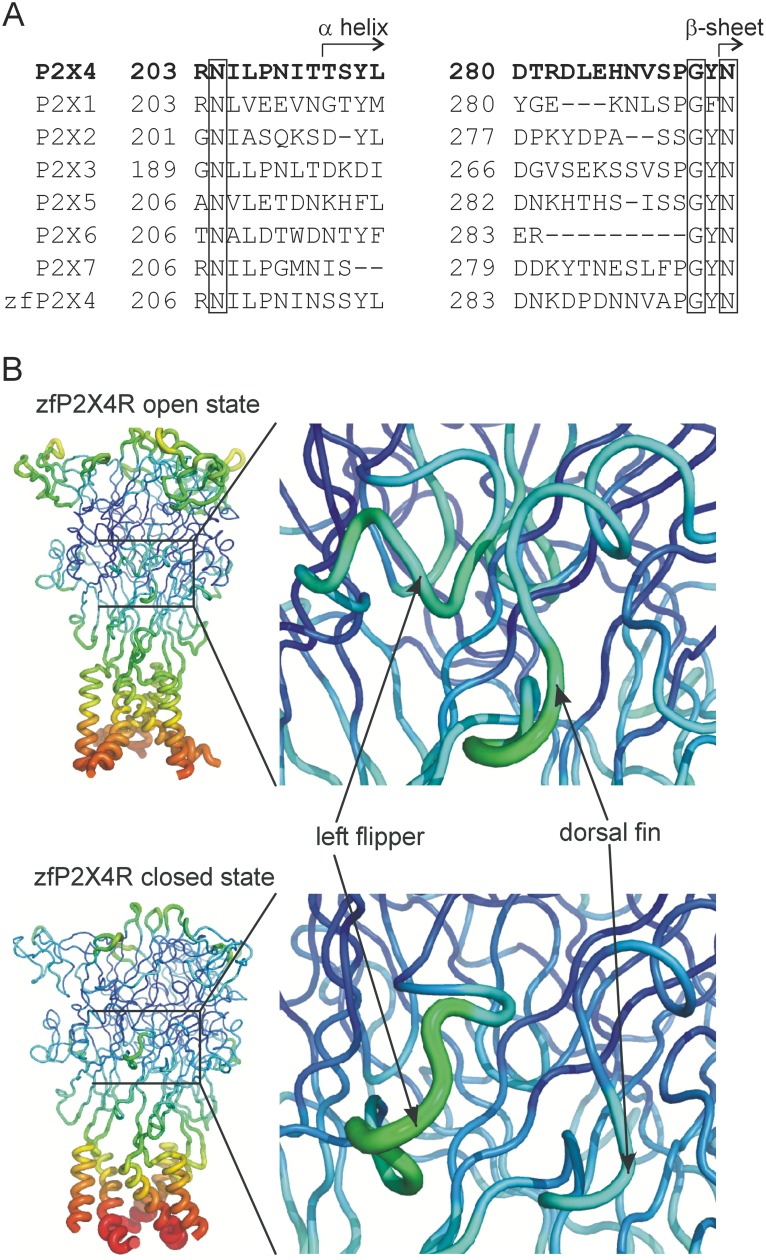
Structural and tridimensional organization of the DF and LF domains. (A) Alignment of amino acid sequences from R203-L214 (DF domain) and D280-N293 (LF domain) using seven rP2X and zfP2X subunits. Structurally, these regions are composed of random coils that terminate with short α-helix and β-sheet structures (indicated by arrows). Conserved amino acid residues are shown in boxes. (B) The models of the zfP2X4.1R are shown in the open (4DW1) or closed (4DW0) state, and the Debye-Waller factor (B-factor) indicates the degree to which the electron density is spread (miniatures). The model shows the elevated B-factor values within the region of intersubunit interaction (magnified segments). Higher B-factors are indicated with thicker cylinders and a red-shifted color, while the lowest B-factors are represented with the thinnest cylinders and a blue-shifted color.

Following ATP binding, the head, upper body, and LF domains of one subunit and the lower body and DF domains of another subunit undergo marked movement that results in the closing of the ATP binding site jaw [11]. During this movement, the LF and DF domains remain in close proximity (Fig. 1B). This promotes expansion of the upper vestibule, leading to the activation of P2XRs [26]. The P2X6R receptor lacks most of the LF domain (Fig. 1A) and is incapable of forming functional homomeric channels [27]. However, it can form functional heteromeric channels with P2X2 and P2X4 subunits [28], [29], which may indicate that one or two complete LF domains per receptor are needed to activate the channel after ATP binding.

Crystallographic data also indicate that the DF and LF domains are intrinsically unfolded and lack secondary structures. These regions have significant conformational flexibility due to higher Debye-Waller factors (B-factors; Fig. 1B). This reflects the thermal fluctuation of atoms in zfP2X4R crystals, as assessed by X-ray scattering techniques, around their average positions and provide important information about protein dynamics [30]. Most importantly, the specific role(s) of non-structural and low-conserved DF and LF regions (Fig. 1A) is not well understood. In particular, we do not know the physiological relevance of having these domains positioned between an ATP binding site and the downstream K313-I333 β-sheet that has been previously identified in rat P2X4R (rP2X4R) as important for transmission of signal from the binding site to the channel gate [31].

We examined the hypothesis that the DF and LF domains may influence the organization of the ATP binding pocket, transmission of ATP-induced signal from ATP binding pocket to the gate, and receptor desensitization. To do this, we used 26 mutants generated by alanine scanning mutagenesis of the R203-L214 (DF) and D280-N293 (LF) sequences. These regions are highly variable between P2XRs, and only few of these residue mutants have been previously characterized electrophysiologically (Table 1). We expressed the wild type (WT) rP2X4R and alanine mutants in HEK293 cells and studied the current responses induced by the application and withdrawal of ATP or its analog agonists 2-(Methylthio)adenosine 5′-triphosphate (2-MeS-ATP), Adenosine 5′-(γ-thio)triphosphate (ATPγS), 2′(3′-O-(4-Benzoylbenzoyl)adenosine 5′-triphosphate (BzATP), and α,β-methyleneadenosine 5′-triphosphate (α,β-meATP), both in the presence and absence of ivermectin (IVM), an allosteric regulator of P2X4R [32]–[34].

**Table 1:** Summary of the changes in estimated EC_50_ values for ATP and changes in desensitization at the DF and LF alanine/cysteine mutants of P2X1-4R residues from published data.

P2X1R	P2X2R	P2X3R	P2X4R
***DF***			
N204A: ↑3,5x^[45]^	N202A: n.f.^[6]^	-	N204A: n.i.
-	-	-	I205A: ↑2,5x^[49]^
-	-	-	L206A: ≈^[49]^
-	-	T196A: ≈^[54]^	T210A: n.i.
-	-	E197A: D^[53]^	T211A: n.i.
-	-	M200C:↑8x^[58]^	L214A: ↑8x^[41]^, 3x^[49]^
***LF***			
-	D277A:: ≈^[56]^	D266A: D^[53]^	D280A ↑>100x^[10], [52]^
		S267A: ≈^[59]^	T281A: n.i.
-	-	S269A: D^[54]^	D283A: n.i.
-	-	-	H286A: ↑2–4x^[10], [52], [57], [60]^
-	-	-	N287A: ≈^[10], [52]^
-	-	-	V288A: ↑2x^[49]^
-	-	S275A: D^[55]^	S289A: n.i.
P287C: ≈^[9]^	-	-	P290A: n.i.
G288A/C: ≈^[47]^, ↑10x^[9]^	-	-	G291A: n.i.
N290A: ↑>50x^[45]^	N288A: ↑>100x^[6]^	N279A: ↑20x^[4]^	N293A: n.i.

n. f., non-functional mutants; ↑ mutant with significant increased EC_50_ in comparison to WT (values represent fold increase); ≈ close to WT receptor; D, affected time-constant of the desensitizing current response; -, non-investigated position; n.i., non-investigated P2X4R mutants that were analyzed in this study.

## Methods

### Cells culture and transfection

To express the recombinant channels, we used human embryonic kidney (HEK) 293T cells (American Type Culture Collection, Rockville, MD, USA) grown in Dulbecco modified Eagle’s medium (Thermo Fisher Scientific, Waltham, MA) supplemented with 10% fetal bovine serum (Sigma-Aldrich, St Louis, MO), 50 U/ml penicillin and 50 µg/ml streptomycin (both Thermo Fisher Scientific, Waltham, MA) in a humidified 5% CO_2_ and 95% air at 37°C. Cells were cultured in 75 cm^2^ plastic culture flasks (NUNC, Rochester, NY) for 36–72 hours until reaching 80–95% confluence. Before the day of transfection, the cells were plated on 35 mm culture dishes (Sarstedt, Newton, NC) and incubated at 37°C for at least 24 h. Transfection was done using 2 µg of either WT or mutant receptor DNA with 2 µl of JetPrime reagent in 2 ml of Dulbecco modified Eagle’s medium, according to manufacturer’s instructions (PolyPlus-transfection, Illkirch, France). Transfected cells were identified by the fluorescence signal of EGFP using the Olympus IX71 inverted research microscope with fluorescence illuminators (Model IX71; Olympus, Melville, NY).

### DNA constructs

cDNAs encoding the sequences of the rP2X4 and mutated subunits were subcloned into the pIRES2-EGFP vector (Clontech, Mountain View, CA, USA). To generate the mutants, oligonucleotides (synthesized and provided by VBC-Genomics, Vienna, Austria or Sigma Aldrich) containing specific mutagenesis mismatches were introduced into the rP2X4/pIRES2-EGFP template using PfU Ultra DNA polymerase (Thermo Fisher Scientific). A High-Speed Plasmid Mini Kit (Geneaid, Taipei City, Taiwan) was used to isolate the plasmids for transfection. Dye terminator cycle sequencing (ABI PRISM 3100, Applied Biosystems, Foster City, CA) was used to identify and verify the presence of the mutations. The sequencing was performed by the DNA Sequencing Laboratory, Institute of Microbiology, ASCR, Prague.

### Patch clamp recordings

ATP-induced currents were recorded from whole cells clamped to −60 mV using an Axopatch 200B patch-clamp amplifier (Axon Instruments, Union City, CA). The recordings were captured and stored using the Digidata 1322A and pClamp9 software package (Axon Instruments). During the experiments, the cell culture was perfused with a bath solution containing: 142 mM NaCl, 3 mM KCl, 2 mM CaCl_2_, 1 mM MgCl_2_, 10 mM 4-(2-Hydroxyethyl)piperazine-1-ethanesulfonic acid (HEPES) and 10 mM D-glucose, adjusted to pH 7.3 with 1 M NaOH. The patch electrodes were filled with a solution containing: 154 mM CsCl, 11 mM EGTA and 10 mM HEPES, adjusted to pH 7.2 with 1.6 M CsOH. The whole-cell configuration was used to abolish the influence of natively present metabotropic receptors for ATP and we used intracellular cesium to block any kind of possible background potassium conductance. Potency of ATP was measured based on the activation of naïve (not previously stimulated) receptors using a short (2–5 s) application of various concentrations of ATP. The results are expressed as molar concentration of ATP required to produce 50% of the maximal response (EC_50_). One or two responses were recorded from one cell, if not otherwise stated, and responses from different cells were pooled. The maximum current amplitude (I_max_) was measured in response to application of supramaximal concentrations (100–1000 µM) of ATP. Responsiveness to P2XR agonists 2-MeS-ATP, ATPγS, BzATP, and α,β-meATP, all applied in 100 µM concentration, was expressed as percentage of response in comparison to 100 µM ATP treatment for selected mutants. In all mutants, the whole cell currents were also measured in the presence of 3 µM IVM, which was dissolved in dimethyl sulfoxide, stored in stock solutions at 10 mM, and diluted to required concentrations in bath solution in the day of experiment. The control and drug containing solutions were applied via a rapid (exchange time 30–40 ms) perfusion system (RSC-200, BIOLOGIC, Claix, France). All other chemicals are from Sigma-Aldrich.

### Calculations

The concentration-response data points were fitted with the equation y = I_max_/[1+ (EC_50_/x)^h^], where *y* is the amplitude of the current evoked by ATP, I_max_ is the maximum current amplitude induced by 100–1000 µM ATP, EC_50_ is the agonist concentration producing 50% of the maximal response, *h* is the Hill coefficient, and *x* is the concentration of ATP (SigmaPlot 2000 v9.01; SPSS Inc., Chicago, IL). Hill coefficient was fixed to 1.3 in all experiments, a value obtained for the WT receptor by fitting. The kinetics of deactivation (current decay evoked by washout of agonist) and desensitization (current decay in the continuous presence of agonist) were fitted by a single exponential function (y = A_1_ exp(−t/τ_1_)) or by the sum of two exponentials (y = A_1_ exp(−t/τ_2_) + A_2_ exp(−t/τ_2_)), respectively, using the program Clampfit 10 (Axon Instruments), where A_1_ and A_2_ are the relative amplitudes of the first and second exponentials, and τ_1_ and τ_2_ are the time constants. The derived time constants for deactivation and desensitization were labeled as τ_off_ and τ_des_, respectively. Weight desensitization constant was calculated as y = [(A_1_τ_des1_) + (A_2_τ_des2_)]/(A_1+_A_2_). Correlation coefficient was calculated using linear regression wizard (SigmaPlot 2000 v9.01). Data points are presented as mean ± SEM values. Significant differences (**p<0.01 and *p<0.05) between means were determined using SigmaStat 2000 v9.01. The data for alanine mutants were analyzed by an ANOVA and Tukey’s post hoc test.

### Homology modeling

The rP2X4R (P51577) and zfP2X4.1R (Q6NYR1) share 61% identity at the amino-acid level, measured with the Basic Local Alignment Search Tool (BLAST; The UniProt Knowledgebase), a value sufficient to build a homology model of the rP2X4R using the automated mode of the SWISS-MODEL server [35]. We extracted a tertiary structure template from the Brookhaven Protein Data Bank under the accession number 4DW0 for the receptor in the apo-closed state and 4DW1 for the zfP2X4.1R in the ATP-bound open state. Model quality was estimated by a SWISSMODEL through a Qualitative Model Energy Analysis (QMEAN) score, which represents a composite scoring function describing the major geometrical aspects of protein structures by taking into consideration five different structural descriptors [36], and which was 0.593. The graphical representations of the protein structure were prepared using PyMOL software (DeLano Scientific LLC, USA).

## Results

### 1. Identification of DF and LF mutants with affected ATP potency and efficacy

**Figure 2:**
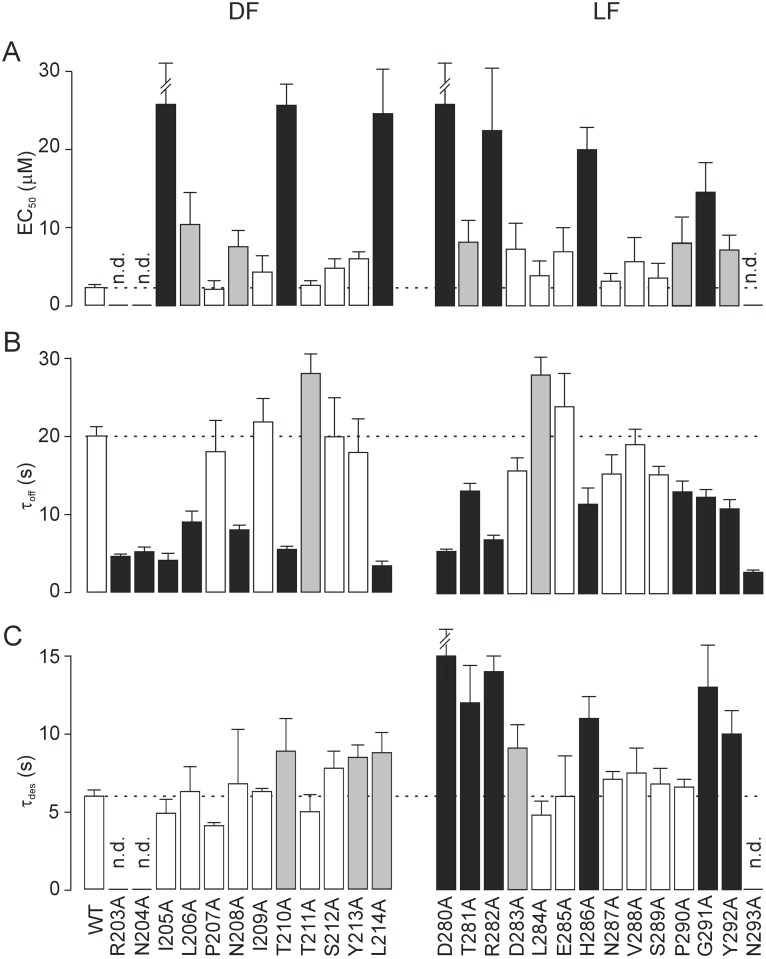
Characterization of rP2X4R–DF and -LF residue mutants. Effect of alanine substitutions on the potency of ATP (A), deactivation (B), and desensitization (C) kinetics. Summary histograms show the concentration of ATP producing a half-maximal current (EC_50_), deactivation time constants (τ_off_) were estimated by the monoexponential fit of the decay of current in response to 2 s of stimulation with 1–3 µM ATP after 4–6 min of preincubation with 3 µM IVM and desensitization time constants (τ_des_) were derived from the biexponential fit of the response to 60 s of stimulation with 100 µM ATP for WT and alanine mutants of the dorsal fin (DF) and the left flipper (LF) domains. Values shown (and also given in Table S1) are the mean ± SEM of 21–63 measurements per mutant and 267 measurements for the WT. Significant differences between the WT and the mutant receptors are shown in gray (p<0.05) or black (p<0.01) columns. Horizontal dotted lines illustrate the values for WT receptor and n.d. indicates that the value could not be determined.

**Figure 3:**
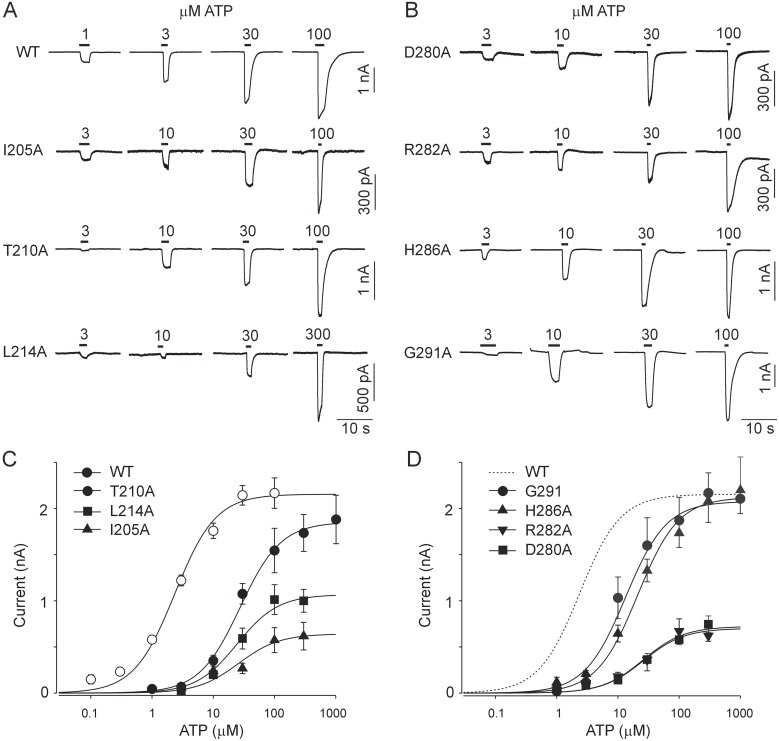
DF and LF mutants exhibit a rightward shift in EC_50_. (A, B) Example records of ATP-induced currents from cells expressing the WT receptor and I205A, T210A, and L214A DF mutants (A) and D280A, R282A, H286A, and G291A LF mutants (B). Currents were stimulated by a short (2–5 s) application of different concentrations of ATP (1–1000 µM), indicated by horizontal bars above the traces. Experiments were performed on naïve receptors, and traces from different cells are shown. (C, D) Concentration response curves for WT, I205A, T210A, and L214A DF mutants (C) and D280A, R282A, H286A, and G291A LF mutants (D). Data points are presented as the mean ± SEM from 7–35 measurements per mutant, per concentration and 78 measurements for WT.

To address the structure-function relationship between the LF and DF regions of rP2X4R, we performed single-point mutagenesis on sequences encompassing the LF and DF regions R203-L214 (DF) and D280-N293 (LF) (Fig. 1A). The crystal structure of the zfP2X4R showed elevated B-factor values in these regions, indicating conformational flexibility (Fig. 1B). For the initial electrophysiological characterization, we examined the EC_50_ and I_max_ values to determine ATP potency and efficacy, respectively. The results from experiments on both mutant and WT receptors are summarized in Figs. 2A and 3 and Table S1.

There were no significant effects on the EC_50_ or I_max_ values for P207A, I209A, T211A, S212A, Y213A, D283A, L284A, E285A, N287A, V288A, and S289A mutant receptors. The EC_50_ values for mutants R203A, N204A, and N293A could not be determined because they displayed a very low ATP-induced current (I_max_ ≤0.2 nA). Mutants I205A, L214A, D280A, R282A, and P290A showed a significant reduction (p<0.01) in I_max_, and with exception of P290A, their EC_50_ values were approximately 10-fold rightward shifted when compared to the WT receptor. The EC_50_ values for T210A, H286A, and G291A mutants were 6- to 10-fold rightward shifted but these receptors showed no significant difference in I_max_ values when compared to the WT receptor (Fig. 3). Slightly less significant increases (p<0.05) in EC_50_ values were observed in the L206A, N208A, T281A, P290A, and Y292A mutants. With the exception of P290A, none of these mutants exhibited significant changes in their I_max_ value. Thus, in 15 of 26 alanine mutants located at the interface of the LF and DF domains the potency and/or efficacy of ATP was significantly reduced, indicating the relevance of these residues in receptor functions.

### 2. IVM rescues the low-functioning DF- and LF-rP2X4R mutants

**Figure 4:**
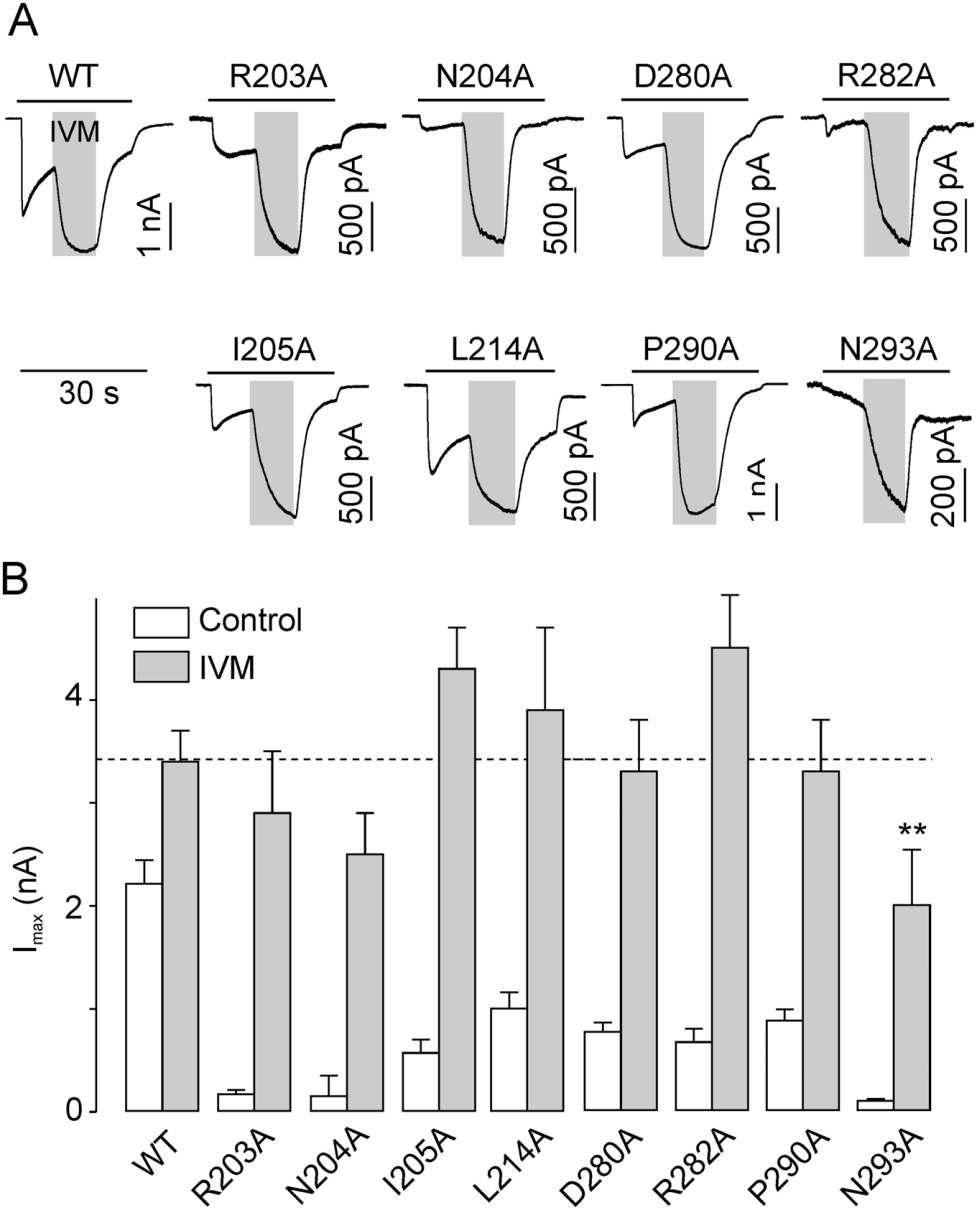
Ivermectin rescues the I_max_ of low-functioning mutants. (A) Acute effect of 3 µM ivermectin (IVM) applied for 10 s (gray areas) during ongoing stimulation with 100 µM ATP for 30 s (horizontal bars) in cells expressing the WT, the DF mutants (R203A, N204A, I205A, and L214A), or the LF mutants (D280A, R282A, P290A and N293A). Recordings are examples of traces similar to 3–5 traces per mutant and 30 per WT receptor. (B) Summary data showing the potentiating effect of IVM preapplication (for 4–6 min) on I_max_ in WT and alanine mutant receptors. The I_max_ values were derived from measurements taken in the absence (open bars) or in the presence (filled bars) of IVM. Values are presented as the mean ± SEM from 5–8 measurements per mutant and 15 measurements per WT. IVM treatment rescued the I_max_ of all low-functioning receptors, except in the case of N293A, which is an ATP binding mutant. The statistical significance was determined by an ANOVA comparing the WT I_max_ and the I_max_ of mutant receptors in the presence of IVM. **, p<0.01.

The effect of IVM on I_max_ was tested initially during ongoing responses to 100 µM ATP to determine whether the low current amplitudes observed in R203A, N204A, I205A, L214A, D280A, R282A, P290A, and N293A mutants could be rescued. The application of 3 µM IVM increased immediately the amplitude of ATP-induced responses in all low-functioning mutants (Fig. 4A). Next, we performed quantitative analysis of I_max_ in WT and all alanine mutants before and after 4–6 min pretreatment with IVM (Table S1). The WT receptor was potentiated 1.5-fold by IVM, while the low-functioning mutants were potentiated 3.7- to 16-fold. In the presence of IVM, the I_max_ values of all low-functioning mutants were comparable to those of WT receptors, except for N293A (Fig. 4B). These experiments indicate that R203, N204, I205, L214, D280, R282, R290, and N293 residues play a critical role in agonist binding and/or channel gating.

The IVM-induced rescue of I_max_ values made it possible to examine the deactivation time constant (τ_off_) for these mutants, which inversely correlates with EC_50_ values [37], [38]. As a result, we were able to more precisely characterize the potency of ATP under comparable conditions. A prolongation of current decay comparable to that observed in WT receptor would suggest that normal ATP potency has been maintained. Alternatively, a decrease or increase in the rate of decay would argue for reduced or enhanced ATP potency, respectively [10]. The deactivation time constant was examined in all alanine mutants by monoexponential fitting of the decay of current after washout of a non-desensitizing concentration of agonist (1 or 3 µM ATP) in the presence of 3 µM IVM. Example traces from WT and mutant receptors with changed deactivations are shown in Fig. S1. The results of τ_off_ measurements are summarized in Fig. 2B and Table S1.

**Figure 5:**
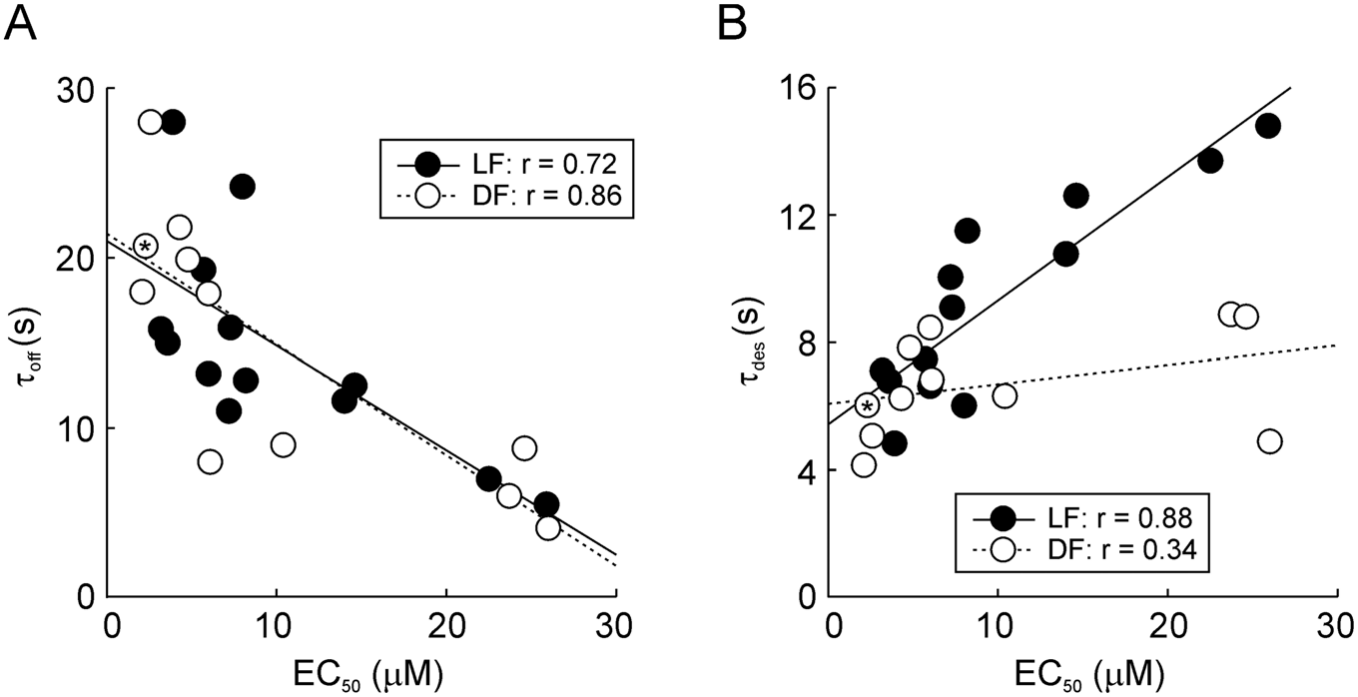
Deactivation and desensitization properties depend on the potency of ATP. (A, B) Correlation between EC_50_ and the deactivation time constant τ_off_ (A) and EC_50_ and desensitization time constant τ_des_ (B) for alanine DF and LF mutants. DF mutants are shown as open circles and LF mutants as closed circles. WT receptors are shown as an asterisk in an open circle. Values are derived from Table S1. Correlation analysis was performed as described in the Methods.

In parallel with the rightward shift changes in EC_50_ values, we observed a significantly (p<0.01) accelerated rate of deactivation in non-responding mutants (R203A, N204A, and N293A) and all rightward shifted mutants (I205A, L206A, N208A, T210A, L214A, D280A, T281A, R282A, H286A, P290A, G291A, and Y292A). Less significant (p<0.05) prolonged deactivation times were found in the L284A and T211A mutants. There was a significant correlation, with highly comparable slopes, between the EC_50_ vs. τ_off_ values for both DF and LF mutants (Fig. 5A). These data confirmed that residues in both domains contribute significantly to receptor activation as well as to receptor deactivation, i.e., that deactivation is a reverse process occurring through the same signal transmission pathway.

### 3. Desensitization kinetics of LF- and DF-rP2X4R mutants

Next, we determined the desensitization kinetics of alanine mutants at the interface of the LF and DF domains. In the presence of 100 µM ATP for 60 s, the WT receptor current declined biexponentially with τ_des1_ = 1.3±0.2 s and τ_des2_ = 9.0±0.7 s; the slow component contributed to the decay with 63±3.0% (Table S2; Fig. S1). The decay of current was also biexponential in all mutants, but in some cases monoexponential fit was the best, and we used a weighted desensitization time constant (τ_des_) for comparison between the mutants and the P2X4R–WT (WT, τ_des_ = 6.0±0.4 s; Table S1 and Fig. 2C). The LF mutants D280A, T281A, R282A, D283A, H286A, G291A, and Y292A exhibited 1.5- to 2.6-fold slower desensitization kinetics when compared to the WT receptor. Less significantly (1.3- to 1.5-fold; p<0.05) prolonged τ_des_ were observed in DF mutants T210A, Y213A, and L214A. The remaining mutants (I205A, L206A, P207A, N208A, I209A, T211A, S212A, L284A, E285A, N287A, V288A, S289A, and P290A) displayed no changes in the desensitization rate. Plotting τ_des_ versus EC_50_ (Fig. 5B) revealed a significant correlation for LF, but not for DF mutants. These results indicate that clusters of residues rather than individual amino acids, are responsible for the desensitization rate of P2X4R and that the LF domain plays a dominant role in this process.

### 4. The influence of the LF and DF domains of rP2X4R on agonist selectivity

To examine whether mutations in the LF and DF domains alter the responsiveness to orthosteric ligands, we compared the efficacy of ATP with four partial agonists for P2X4R. In WT receptor, 100 µM was maximal concentration for all analogue agonists, except α,β-meATP (2-MeS-ATP, EC_50_ = 7.9±1.0 µM; ATPγS, EC_50_ = 8.4±1.8 µM; BzATP, EC_50_ = 11.1±2.9 µM; α,β-meATP, EC_50_ = 62±18 µM; Fig. S2A, *upper panel*) and the agonist efficacy profile of the WT receptor was ATP (100%) >2-MeS-ATP (67%) > ATPγS (50%) > BzATP (38%) > α,β-meATP (32%). We examined all of the functional mutants that displayed significant changes in ATP potency and/or deactivation kinetics (I205A, L206A, N208A, T210A, T211A, L214A, D280A, T281A, R282A, L284A, H286A, P290A, G291A, and Y292A) and two substitution-insensitive mutants (S212A, Y213A) (Table 2).

**Table 2:** The relative responsiveness of the wild type (WT) and selected rP2X4R mutants to P2XR agonist analogs.

Receptor	Domain	2-MeS-ATP	ATPγS	BzATP	α,β-meATP
**WT**		67.3±4.6	49.6±3.5	38.2±3.4	31.8±3.3
*Group I*					
**I205A**	DF	35.6±1.9**	11.3±2.2**	4.4±2.6**	4.6±1.0**
**T210A**	DF	59.2±6.9	34.2±4.1*	23.9±3.2**	8.8±2.3**
**L214A**	DF	21.4±1.7**	7.9±2.0**	5.9±0.5**	6.5±1.5**
**P290A**	LF	50.7±3.7	37.0±11.0	16.9±3.2**	17.8±2.4**
**G291A**	LF	36.9±8.8**	31.6±6.1*	53.0±4.0**	2.8±0.5**
**Y292A**	LF	49.8±8.6	42.3±7.0	28.9±8.2	16.2±4.4**
*Group II*					
**L206A**	DF	68.3±6.5	39.6±8.4	33.7±8.0	27.5±2.5
**N208A**	DF	60.0±11.2	42.2±5.2	33.4±5.4	28.5±2.3
**D280A**	LF	56.3±7.8	55.7±3.8	25.4±1.5	33.1±6.0
**T281A**	LF	69.6±5.1	50.2±5.9	26.7±3.9	28.2±5.5
**R282A**	LF	55.1±12.9	45.5±5.2	25.7±3.7	27.3±7.9
**H286A**	LF	67.9±1.2	39.8±6.2	25.6±4.0	30.0±6.8
*Group III*					
**S212A**	DF	65.1±19.7	61.7±3.9	47.5±5.2	19.2±6.2
**Y213A**	DF	63.8±16.4	37.1±5.5	27.3±5.5	31.3±6.7
**T211A**	DF	79.3±6.7	61.8±6.1	23.4±4.7*	39.1±7.2
**L284A**	LF	69.0±3.0	32.4±8.2	20.6±0.2**	22.2±3.9

ATP and agonists were applied in 100 µM concentrations for 2 s with a washing interval of 60 s. The data are the mean ± SEM, relative to ATP efficacy (100%), from 26 to 37 measurements for the WT receptor and from 3 to 18 measurements per mutant. *Group I:* Mutants that exhibited changes in ATP potency/efficacy, deactivation kinetics, and changes in the relative responsiveness to orthosteric analog agonists. *Group II:* Mutants that exhibited changes in ATP potency/efficacy and deactivation, but no changes in the relative responsiveness to analog agonists. *Group III:* Mutants that showed no significant changes in ATP potency/efficacy. The statistical significance was determined by an ANOVA comparing the responsiveness to agonists between WT and mutant receptors: **, p<0.01, *, p<0.05. DF, Dorsal Fin; LF, Left Flipper.

The mutants could be divided into one of three groups. The *Group I* was composed of mutants with changes in ATP potency/efficacy and deactivation kinetics that also exhibited a significant decrease in the relative responsiveness to some or all of the orthosteric agonists applied in 100 µM concentration (Table 2, Fig. S2B). This group includes I205A, T210A, L214A, P290A, G291A, and Y292A mutants. The I205A and L214A mutants displayed a significantly reduced responsiveness to all agonists when compared to the WT receptor, and the profile (2-MeS-ATP > ATPγS > BzATP ≥ α,β-meATP) was preserved (Table 2). This suggests that these residues also play a role in the mechanism by which an ATP-induced signal is coupled to the channel gating. In contrast, there were changes in the agonist profile for the T210A, P290A, G291A, and Y292A mutants. For example, the profile for the G291A mutant was BzATP > 2-MeS-ATP > ATPγS > α,β-meATP (Fig. S2A, *lower panel*), indicating changes in the folding of the jaw for ATP.

The *Group II* of mutants showed changes in ATP potency/efficacy and deactivation, but not in the relative responsiveness to orthosteric agonists to induce current. This includes the L206A, N208A, D280A, T281A, R282A, and H286A mutants (Table 2). The four members of *Group III*, T211A, S212A, Y213A, and L284A, showed no significant changes in ATP potency/efficacy or gating, and among them, only T211A and L284A displayed slightly (p<0.05) prolonged deactivation times (Fig. 2B). These two mutants also showed a significant decrease in the responsiveness to BzATP but no decrease in the responsiveness to 2-MeS-ATP, ATPγS, and α,β-meATP (Table 2). Therefore, residues T211 and L284 were not considered as residues of interest.

### 5. Model prediction for the positions of residues of interest in the DF and LF domains

**Figure 6:**
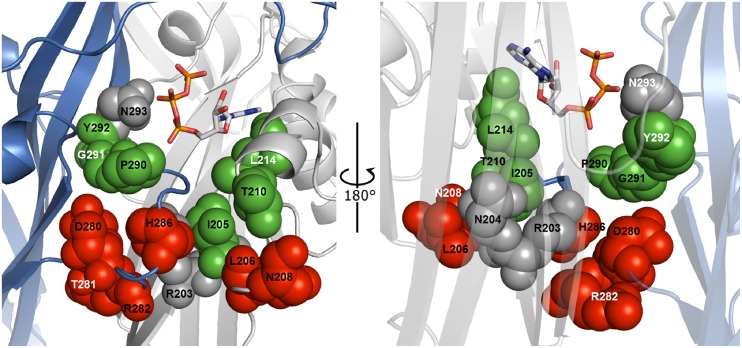
The structure of the ATP binding site in a rP2X4R homology model. Two panels show the position of affected residues (rotated 180°) at the interface between the LF and DF domains. The low-response residues without defined EC_50_ values are grey spheres. The amino acid residues presented as green spheres demonstrate the topology of mutants with changes in ATP potency and/or efficacy (EC_50_ and I_max_), and agonist profile (*Group I* from Table 2). The amino acids presented in red spheres illustrate the position of residues whose mutation has affected ATP potency and/or efficacy without changing the action of ATP analogs (*Group II* from Table 2). In both panels, the ATP molecule is situated between two adjacent P2X4R subunits (blue and gray). The ATP molecule is shown in a wireframe model.

We developed the rP2X4R homology model, as described in Materials and Methods, to identify the position of residues in the DF and LF domains in the ATP-bound open state. The data presented in Table 2 are also summarized in Fig. 6 as a receptor structure view. Native residues from the *Group I* mutants are located close to the ATP molecule, near the N293 residue. All of the residues from the *Group II* mutants are located downstream of the ATP binding domain and near the R203 and N204 residues that are burrowed in the protein. This topology suggests that the I205, T210, L214, P290, G291 and Y292 (green spheres) contribute to the organization of the structure of the ATP binding pocket and therefore dictate the specificity of responsiveness to the synthetic orthosteric ligands as well as the transmission of the conformational change induced by ATP binding. Residues L206, N208, D280, T281, R282, and H286 (red spheres) are important for transmitting the signal from the ATP binding cleft. Residues of mutants that have shown significant gating impairment (R203, N204 and N293) are shown as gray spheres.

The N293 residue is in close proximity (less than 5 angstroms) to ATP and, together with Y292, may directly interact with the β-sheet segment of the K313-I333 sequence, which is responsible for the signal transmission from the ATP binding site to the pore [31] (Fig. S3A). The R203 and N204 residues are situated at the bottom of the ATP binding site (Fig. S3B) and play a crucial role in the transmission of signals towards the pore. The model also indicates that residues D283, H286, V288, and S289, from the adjacent subunit, are in the proximity of R203, while the K190, N191, N204, I205, and L206 residues are in close proximity within the same subunit. Close residues for N204 (I205, L206, and Y274) are also located within the same subunit (Fig. S3B). This suggests that the R203 and N204 residues may integrate the output signal from two neighboring subunits towards the gate and their mutants may display radical conformational misfolding of the DF and LF domains.

## Discussion

The interface between the DF and LF domains, formed by sequences R203-L214 and D280-N293 in rP2X4R, is one of the most variable parts of the P2XRs [12]. Alignments of these regions for seven rat P2X subunits indicate that only three of 26 amino acids are fully conserved (N204, G291, and N293). Four hydrophobic residues, at positions I205, L206, L214, and V288, are partially conserved, and the residual amino acids of this interface are variable (Fig. 1A). Such variability in the structure of the DF and LF domains could indicate that they are not essential for receptor function or that they contribute to receptor subtype specificity in terms of agonist binding and/or gating, and desensitization.

In this study, the physiological relevance of the residues that comprise the DF and LF domains of rP2X4R was systematically analyzed for the first time by substituting each residue with an alanine. This approach enabled us to eliminate interactions between the side chains and to study the effects of that elimination on rP2X4R structure and activity. Alanine was also used as a substituent because its polarity is in the middle of the polarity scale [39] when compared to other residues. Moreover, alanine scanning mutagenesis has been widely used in research on P2XRs [6], [18], [23], [40].

Electrophysiological and pharmacological characterization of the mutants revealed that substitution of 15 of 26 residues in the R203-L214 and D280-N293 sequences significantly attenuated the receptor function: R203A, N208A, T210A, T281A, R282A, P290A, G291A and Y292A, not previously studied, and N204A, I205A, L206A, L214A, D280A, H286A and N293A, previously studied across different P2XR subtypes (for overview see Table 1). However, the receptor function for all identified mutants, including almost non-functional mutants R203A, N204A and N293A, was rescued by the addition of IVM, an allosteric agonist of P2X4R [41].

In general, IVM allosterically potentiates the I_max_ of P2X4R, causes a leftward shift in the ATP concentration response curve and significantly prolongs deactivation [32], [33]. Single channel analysis showed that IVM increases the probability of channel opening [34]. We have recently found that IVM induces dilation of the pore of the P2X4R ion channel and that the IVM-dependent transition from open to dilated state temporally coincides with receptor sensitization, which rescues the receptor from desensitization and subsequent internalization [42]. This suggests that the observed increase in the number of cell surface P2X4Rs after 2–30 min of preincubation with IVM [43] is not due to insertion of new receptors to the plasma membrane, but rather reflects its influence on channel pore dilation. The use of P2X4-pHluorin123 also revealed that IVM does not acutely increase the fraction of P2X4Rs in the plasma membrane [44]. Therefore, it is reasonable to conclude that the trafficking of mutant receptors is not affected, i.e., they are expressed at the plasma membrane, but ATP has reduced binding affinity and/or potency to activate them.

In two of three nonfunctional mutants, N204A and N293A, alanine substitutes conserved asparagine residue. The N293 amino acid was previously identified in the crystal structure of zfP2X4R as an ATP binding residue. This residue is a part of the NFR motif, which is important for the recognition of the triphosphate moiety of ATP [11]. In our experiments, the I_max_ of N293A could not be fully rescued by IVM, similar to previous observations of two other P2X4R ATP binding mutants, K67A and R295A [10]. A decrease in agonist potency was also observed in the corresponding N293A mutants of other receptors, including P2X1R-N290A [45], P2X2R-N288A [6], and P2X3R-N279A [4]. These studies further support the importance of N293 residue in the formation of the ATP binding pocket. In further agreement with our data, mutation of the conserved N204 residue is nonfunctional in P2X1R [6] and causes a 3-fold decrease in ATP potency in P2X2R [45]. An arginine in the position equivalent to 203 is present in P2X1R and P2X7R, and a lysine substitution of residue R206 enhances the sensitivity of P2X7R to activation by ATP [46].

The 12 mutants were functional but exhibited significant changes in the EC_50_, I_max_, τ_off_, and/or τ_des_ values. These mutants were divided into two groups based on the relative responsiveness to stimulation with ATP and four P2XR agonist analogs. *Group I* is composed of mutants with altered relative response to the agonist analogs and includes the I205A, T210A, L214A, P290A, G291A, and Y292A mutants. In contrast, *Group II* mutants showed no change in the responsiveness to analogs and includes the L206A, N208A, D280A, T281A, R282A, and H286A mutants (Table 2). The model prediction for the positions of these residues supports the conclusions that *Group I* residues contribute to the formation of the large ATP binding pocket in addition to signal transmission, while *Group II* residues contribute to signal transmission only. Therefore, both the DF and LF domain residues participate significantly in receptor function.

In our receptor model, in close proximity to the bound ATP molecule and asparagine 293, are the Y292, G291, and P290 residues. The substitution of these residues with alanine altered the ATP potency and/or efficacy, deactivation kinetics, and agonist profile. Among them, the most affected was the G291A mutant that exhibited an approximately 6-fold rightward shifted EC_50_ value and large changes in agonist selectivity profile. Glycine 291 is conserved in all rat P2XRs (Fig. 1A), and the corresponding cysteine mutant of P2X1R showed a 10-fold decrease in ATP potency [9], while the alanine mutant had little effect [47]. However, its role in ligand selectivity and ATP potency in other P2XR subtypes remains to be determined. These data, combined with the topology of residues in the receptor model, suggest that the N293-P290 sequence forms part of a wall in the ATP binding cleft and contributes to signal transmission through downstream LF domain residues (Fig. 6). The model predicts that this segment will also act on the transmission of signals to the gate, possibly by interactions with the K313-I333 β-sheet (Fig. S3A). Consistent with this hypothesis, mutagenesis of the Y315 and G316 residues significantly affects receptor function [31].

The partially conserved hydrophobic residue L214 has also been implicated in the recognition of the ATP ribose ring [11], [48], which is fully consistent with our data. We observed that the L214A mutant displays full recovery of I_max_ in the presence of IVM, and has reduced responsiveness to all orthosteric agonists. However, the agonist profile of the WT receptor was preserved. The DF mutants I205A and T210A also exhibited a reduced potency/efficacy for ATP and its analogs. Topologically, these native residues may account for the bottom part of the ATP binding pocket (Fig. 6). A recent study on hydrophobic interactions between the LF and DF domains during receptor activation has identified several non-polar residues, including L214 and I205, that are important for the coordinated relative movements of these domains after ATP binding [49]. Therefore, we suggest that L214 residue plays a dual role in receptor functions: agonist binding and signal transmission.

The topology of T210 in zfP2X4R revealed that the residue is situated nearby the α-helix containing L214 involved in ATP recognition [11], (Fig. 1A). We observed changes in agonist profile for T210A mutant, suggesting that T210 could contribute to coordination of agonist position in the binding cleft. This explanation needs assumption that the T210 side chain position is variable and might be oriented towards the binding pocket, similarly as L214, and that orientation of ATP is different from that predicted by crystal, suggesting the existence of several ATP binding modes [50], [51]. Further experiments are needed to explain the role of T210 in receptor function.

The homology model of rP2X4R predicts that the *Group II* amino acids are clustered into two subgroups: one composed of D280-H286 LF residues and the other composed of L206-N208 DF residues. The position of these residues is consistent with their roles in signal transmission. Fig. 6 suggests that the influence of ATP binding on gating is transmitted downstream through two signal transmission lines. The first is composed of N293, Y292, G291, and P290 towards D280, T281, R282, and H286 (from top to bottom) in the LF domain. The other unit appears to be composed of L214, T210, I205, N204, L206, and N208 (from top to bottom) in the DF domain. The model also suggests that R203 and N204 are positioned to accept the signal from the binding domain through both lines and from two neighboring subunits, and to integrate it towards the gate region.

Finally, seven out of the 13 LF mutants tested showed significantly slower rates of receptor desensitization and our correlation analysis of the relationship between EC_50_ vs. τ_des_ suggests that that the LF domain plays the major role in the transition from the open to the desensitized state, with signal transmission through the N293-D280 sequence (Fig. 2C and 5B). Alanine substitution of the corresponding positions D266A [53], S269A [54], but also S275A [55], prolongs desensitization of P2X3R, but the P2X2R-D277A mutant was normal [56]. These data indicate that this group of polar and charged residues might play a receptor-specific role in desensitization.

In conclusion, we have shown that the interface between DF and LF domains has dual roles in rP2X4R function. One role is the formation of the ligand-binding pocket and the other is for the transmission of signals from the pocket toward the gate. Both domains contribute to the specificity of binding sites for orthosteric agonists by residues in the upper part of interface, relative to distance from the channel pore, and to the transmission of signals towards the gate by residues in the lower part of the interface. The R203 and N204 may integrate the influence of both lines of transmission. The LF domain appears to have two additional roles: the transmission of signals towards the gate in the second transmembrane domain through the K313-I333 β-sheet and the control of desensitization of receptors.

## Supporting Information

Figure S1 **Deactivation and desensitization responses of WT and selected DF and LF mutants.** (A) An example of the WT response and that of the L214A, D280A, and H286A mutant receptors when stimulated with 3 µM ATP for 2 s in the presence of IVM. Cells were preincubated with 3 µM IVM for 4–6 min, and the deactivation time constants (τ_off_) were estimated by the monoexponential fit of decay of current after removal of the agonist. (B) The desensitization of WT, L214A, D280A, and H286A receptors when stimulated with 100 µM ATP for 60 s (gray traces), and the curves obtained by fitting (black). Weighted desensitization time constants (τ_des_) were derived from monoexponential (D280A) or biexponential fitting.
(TIF)

Figure S2 **Responsiveness of the WT and mutant receptors to ATP analogue agonists.** (A) Concentration response curves for WT and G291A receptors. Currents were stimulated by a short (2–5 s) application of different (1–300 µM) concentrations of ATP, 2-MeS-ATP (2MeS), ATPγS, BzATP (Bz) and α, βme-ATP (αβme). Even if the full dose response curve for analogue agonists could not be constructed for G291A, these experiments clearly show differences in agonist profile between the WT (*upper panel*) and G291A (*lower panel*) receptor. Experiments were performed on naïve receptors, and data points are presented as the mean ± SEM from 3–27 measurements per agonist, per concentration for both WT and G291A. (B) Example responses to 100 µM of ATP and several P2XR agonists, including 2-MeS-ATP (2MeS), ATPγS, BzATP (Bz) and α, βme-ATP (αβme), recorded from cells expressing the WT receptor and selected mutant receptors from from *Group I* (I205A, T210A, L214A, and G291A) and *Group II* (D280A and H286A). Each trace represents a continuous response from a single cell.
(TIF)

Figure S3 **The structure of the ATP binding site in the rP2X4R homology model.** (A) The possible interaction of N293 and Y292 residues with Y315 and G316 residues from the β-sheet segment from the K313-I333 sequence. (B) The multiple interactions of residues R203 (yellow spheres) and N204 (green spheres) with partners (all in cyan wireframes) from the same (K190, N191, N204, I205, L206, Y274) and adjacent (D283, H286, V288, S289) subunits. Two adjacent rP2X4R subunits are represented in blue and gray.
(TIF)

Table S1 Characterization of the DF and LF alanine mutants of rP2X4R.
(DOC)

Table S2 Desensitization parameters for the DF and LF mutants of the rP2X4R.
(DOC)
