# Supplementary figures and images for: Identification of Functionally Important Residues of the Rat P2X4 Receptor by Alanine Scanning Mutagenesis of the Dorsal Fin and Left Flipper Domains
Source: PLoS One. 2014 Nov 14;9(11):e112902. doi: 10.1371/journal.pone.0112902 (PMC4232510; doi:10.1371/journal.pone.0112902)

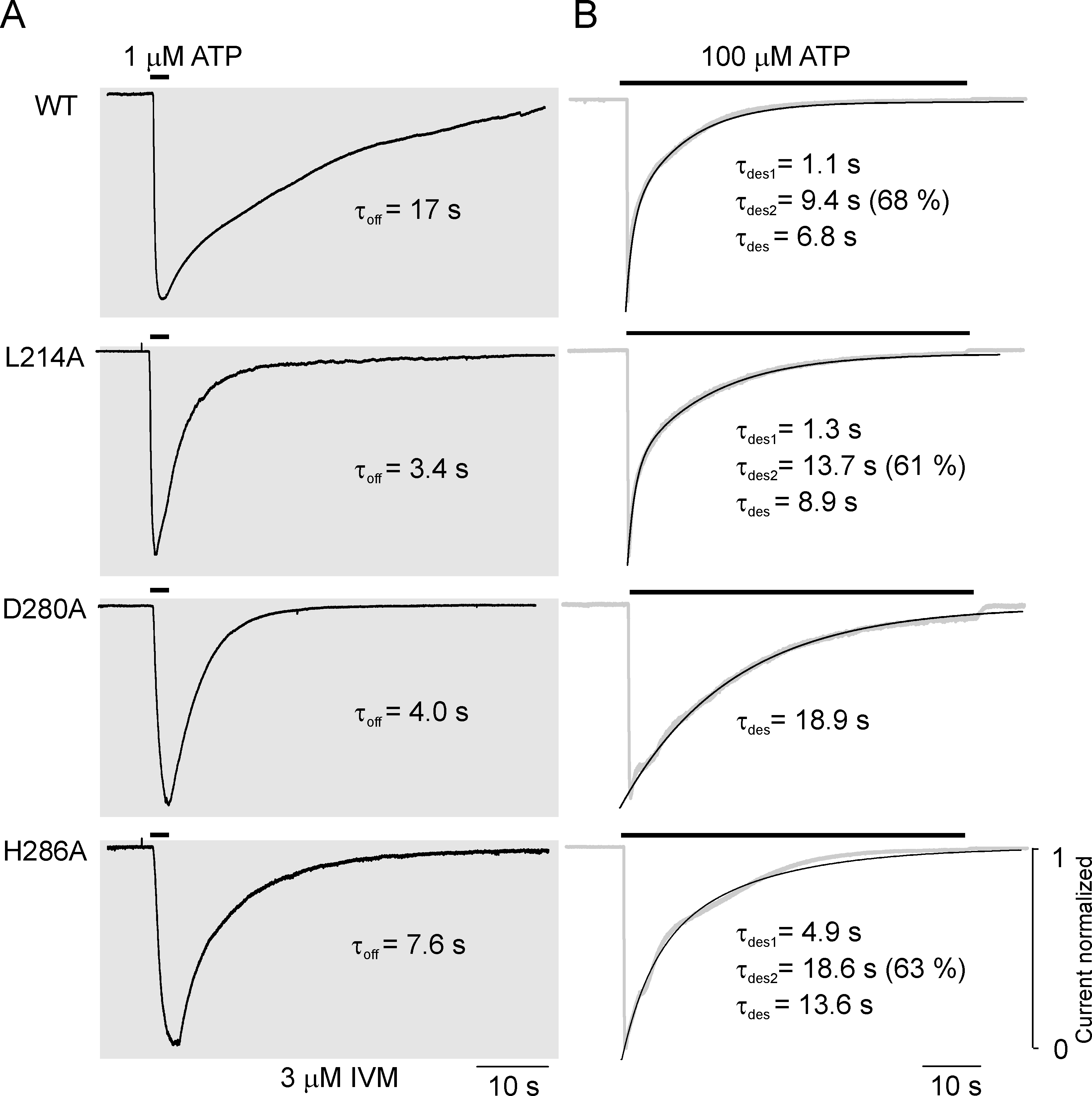

Supplement: Figure S1 — Deactivation and desensitization responses of WT and selected DF and LF mutants. (A) An example of the WT response and that of the L214A, D280A, and H286A mutant receptors when stimulated with 3 µM ATP for 2 s in the presence of IVM. Cells were preincubated with 3 µM IVM for 4–6 min, and the deactivation time constants (τoff) were estimated by the monoexponential fit of decay of current after removal of the agonist. (B) The desensitization of WT, L214A, D280A, and H286A receptors when stimulated with 100 µM ATP for 60 s (gray traces), and the curves obtained by fitting (black). Weighted desensitization time constants (τdes) were derived from monoexponential (D280A) or biexponential fitting. (TIF) [file pone.0112902.s001.tif]

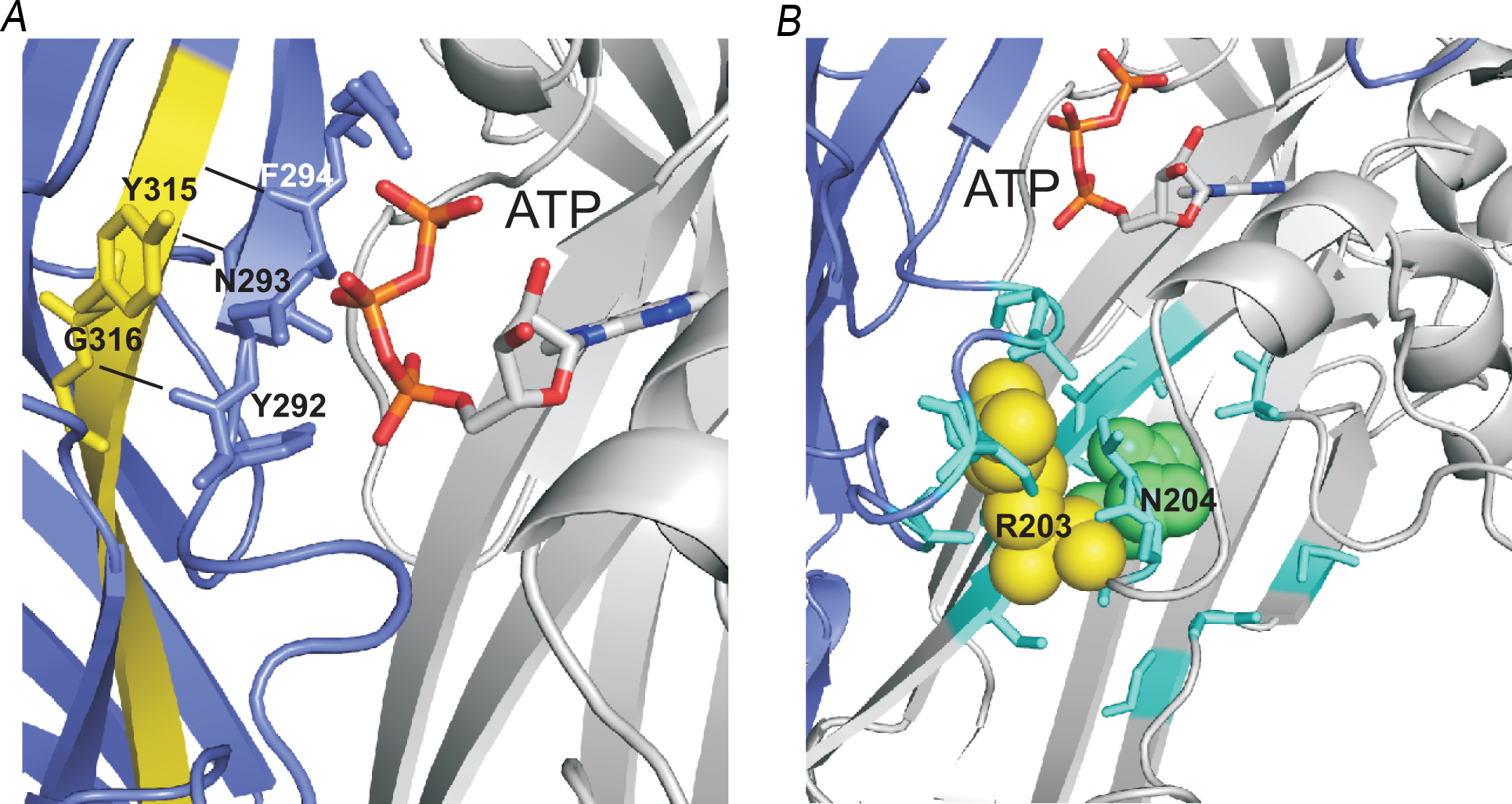

Supplement: Figure S3 — The structure of the ATP binding site in the rP2X4R homology model. (A) The possible interaction of N293 and Y292 residues with Y315 and G316 residues from the β-sheet segment from the K313-I333 sequence. (B) The multiple interactions of residues R203 (yellow spheres) and N204 (green spheres) with partners (all in cyan wireframes) from the same (K190, N191, N204, I205, L206, Y274) and adjacent (D283, H286, V288, S289) subunits. Two adjacent rP2X4R subunits are represented in blue and gray. (TIF) [file pone.0112902.s003.tif]
