# Supplementary material for: Identification of Functionally Important Residues of the Rat P2X4 Receptor by Alanine Scanning Mutagenesis of the Dorsal Fin and Left Flipper Domains
Source: PLoS One. 2014 Nov 14;9(11):e112902. doi: 10.1371/journal.pone.0112902 (PMC4232510; doi:10.1371/journal.pone.0112902)
Supplement: Table S1 — Characterization of the DF and LF alanine mutants of rP2X4R. (DOC) [file pone.0112902.s004.doc]

## Table S1: Characterization of the DF and LF alanine mutants of rP2X4R.

| **Receptor** | **EC_50_** | **I_max_** | **I_max_+IVM** | **Fold incr.** | **τ_off_ +IVM** | **τ_des_** |
| --- | --- | --- | --- | --- | --- | --- |
|  | **[µM]** | **[nA]** | **[nA]** | **I_max_** | **[s]** | **[s]** |
| **WT-P2X4** | 2.3 ± 0.4 | 2.2 ± 0.2 | 3.4 ± 0.3 | 1.7 ± 0.2 | 21 ± 1.0 | 6.0 ± 0.4 |
|  |  |  | ***DF*** |  |  |  |
| **R203A** | n.d. | 0.2 ± 0.1** | 2.9 ± 0.6 | 16 ± 5.0** | 4.6 ± 0.3** | n.d. |
| **N204A** | n.d. | 0.2 ± 0.2** | 2.5 ± 0.4 | 15 ± 5.0** | 5.2 ± 0.6** | n.d. |
| **I205A** | 26 ± 7.7** | 0.6 ± 0.13** | 4.3 ± 0.4 | 5.5 ± 0.6** | 4.1 ± 0.9** | 4.9 ± 0.9. |
| **L206A** | 10 ± 4.1* | 2.4 ± 0.3 | 4.8 ± 0.8 | 1.7 ± 0.2 | 9.0 ± 1.4** | 6.3 ± 1.6 |
| **P207A** | 2.1 ± 1.1 | 2.2 ± 0.2 | 3.3 ± 0.5 | 1.5 ± 0.3 | 18. ± 4.0 | 4.1 ± 0.2 |
| **N208A** | 7.6 ± 2.1* | 1.7 ± 0.3 | 3.6 ± 0.3 | 2.3 ± 0.5 | 9.1 ± 0.2** | 6.8 ± 3.5 |
| **I209A** | 4.3 ± 2.1 | 1.9 ± 0.4 | 4.1 ± 0.8 | 2.1 ± 0.3 | 22 ± 3.0 | 6.3 ± 0.2 |
| **T210A** | 26 ± 2.6** | 1.9 ± 0.2 | 3.5 ± 0.6 | 1.8 ± 0.2 | 5.5 ± 0.4** | 8.9 ± 2.1* |
| **T211A** | 2.6 ± 0.6 | 2.3 ± 0.3 | 3.9 ± 0.7 | 1.6 ± 0.3 | 28 ± 2.5* | 5.0 ± 1.1 |
| **S212A** | 4.8 ± 1.2 | 2.1 ± 0.2 | 3.4 ± 0.2 | 1.6 ± 0.2 | 20 ± 5.0 | 7.8 ± 1.1 |
| **Y213A** | 6.0 ± 0.9 | 2.2 ± 0.4 | 3.1 ± 0.2 | 1.4 ± 0.1 | 18 ± 4.3 | 8.5 ± 0.8* |
| **L214A** | 25 ± 5.7** | 1.0 ± 0.2** | 3.9 ± 0.8 | 3.7 ± 0.7** | 3.4 ± 0.6** | 8.8 ± 1.3* |
|  |  |  | ***LF*** |  |  |  |
| **D280A** | 26 ± 8.2** | 0.7 ± 0.1** | 3.6 ± 0.4 | 5.1 ± 0.5** | 5.5 ± 0.3** | 15 ± 3.7** |
| **T281A** | 8.2 ± 2.8* | 2.3 ± 0.3 | 4.8 ± 0.9 | 2.1 ± 0.2 | 13 ± 1.0** | 12 ± 2.4** |
| **R282A** | 23 ± 8.0** | 0.7 ± 0.1** | 4.5 ± 0.5 | 9.6 ± 2.5** | 7.0 ± 0.6** | 14 ± 1.0** |
| **D283A** | 7.3 ± 3.4 | 2.2 ± 0.3 | 3.1 ± 0.3 | 1.4 ± 0.1 | 16 ± 1.7 | 9.1 ± 1.5* |
| **L284A** | 3.9 ± 1.9 | 2.4 ± 0.3 | 4.1 ± 0.3 | 1.7 ± 0.1 | 28 ± 2.3* | 4.8 ± 0.9 |
| **E285A** | 7.1 ± 3.0 | 2.0 ± 0.5 | 2.6 ± 0.6 | 1.3 ± 0.4 | 24 ± 4.3 | 6.0 ± 2.6 |
| **H286A** | 20 ± 2.9** | 2.2 ±0 .4 | 4.2 ± 0.7 | 1.9 ± 0.3 | 12 ± 2.1** | 11 ± 1.4** |
| **N287A** | 3.2 ± 1.0 | 1.6 ±0.2 | 4.0 ± 0.6 | 3.3 ± 0.4 | 16 ± 2.5 | 7.1 ± 0.5 |
| **V288A** | 5.7 ± 3.1 | 1.3 ± 0.2 | 3.0 ± 0.7 | 2.3 ± 0.3 | 19 ± 2.0 | 7.5 ± 1.6 |
| **S289A** | 3.6 ± 1.9 | 1.4 ± 0.2 | 4.5 ± 1.1 | 3.2 ± 0.2 | 15 ± 1.1 | 6.8 ± 1.0 |
| **P290A** | 8.0 ± 3.4* | 0.9 ± 0.1** | 3.3 ± 0.5 | 3.7 ± 0.2 | 13 ± 1.4** | 6.6 ± 0.5 |
| **G291A** | 15 ± 3.8** | 2.1 ± 0.2 | 3.3 ± 0.4 | 1.4 ± 0.02 | 13 ± 1.0** | 13 ± 2.7** |
| **Y292A** | 7.2 ±1.9* | 1.9 ± 0.3 | 4.3 ± 0.3 | 2.0 ± 0.3 | 11 ± 1.2** | 10 ± 1.5** |
| **N293A** | n.d. | 0.1 ± 0.02** | 2.0 ±0.5** | 14 ± 1.1** | 2.8 ± 0.2** | n.d. |

## Each receptor was examined for potency (EC_50_) and efficacy (I_max_) in response to ATP. The I_max_ was tested with two concentrations of ATP, 100 and 300 μM, except mutants L214A, H286A and G291A that were tested also with 1 mM concentrations of ATP. Mutants were treated with 3 μM IVM for 4-6 min (I_max_+IVM), and the potentiating effect of IVM is expresses as fold increase in the I_max_ value when compared to the IVM-untreated cells (Fold incr. I_max_). The deactivation time constant (τ_off_) was measured by the monoexponential fitting of current decay after removal of 1 or 3 μM ATP in the presence of IVM. The desensitization time constant (τ_des_) was measured by biexponential fitting of current traces in response to the application of 100 μM ATP for 60 s. The data are the mean ± SEM from 21 to 63 measurements per mutant and 267 measurements for the wild type (WT) receptor. DF, Dorsal Fin domain; LF, Left Flipper domain. The statistical significance was estimated by an ANOVA comparing the WT and mutant receptors, p < 0.01 (**), p < 0.05 (*). n.d., values could not be determined.
