# Supplementary material for: Identification of Functionally Important Residues of the Rat P2X4 Receptor by Alanine Scanning Mutagenesis of the Dorsal Fin and Left Flipper Domains
Source: PLoS One. 2014 Nov 14;9(11):e112902. doi: 10.1371/journal.pone.0112902 (PMC4232510; doi:10.1371/journal.pone.0112902)
Supplement: Table S2 — Desensitization parameters for the DF and LF mutants of the rP2X4R. (DOC) [file pone.0112902.s005.doc]

## Table S2. Desensitization parameters for the DF and LF mutants of the rP2X4R.

| **Receptor** | **τ_des1_** | **τ_des2_** | **A_2_ /(A_1+_A_2_)** |  |
| --- | --- | --- | --- | --- |
|  | **[s]** | **[s]** | **[%]** | **n** |
| **WT-P2X4** | 1.3 ± 0.2 | 9.0 ± 0.7 | 63 ± 3 | 31 |
|  |  | ***DF*** |  |  |
| **R203A** | n.d. | n.d. | n.d. | n.d. |
| **N204A** | n.d. | n.d. | n.d. | n.d. |
| **I205A** | 1.5 ± 0.7 | 8.1 ± 1.3 | 51 ± 4 | 4 |
| **L206A** | 1.1 ± 0.2 | 8.9 ± 2.2 | 70 ± 4 | 9 |
| **P207A** | 0.7 ± 0.1 | 5.6 ± 0.2 | 71 ± 2 | 3 |
| **N208A** | 1.1 ± 0.8 | 8.7 ± 4.8 | 77 ± 4 | 5 |
| **I209A** | 2.1 ± 0.8 | 9.3 ± 2.6 | 70 ± 2 | 4 |
| **T210A** | 2.0 ± 0.3 | 14.4 ± 2.5** | 52 ± 8 | 5 |
| **T211A** | 0.9 ± 0.4 | 8.3 ± 2.4 | 60 ± 8 | 4 |
| **S212A** | 1.7 ± 0.4 | 11.4 ± 1.5 | 62 ± 5 | 12 |
| **Y213A** | 3.0 ± 0.3* | 12.4 ± 1.6 | 61 ± 6 | 6 |
| **L214A** | 1.9 ± 0.4 | 12.6 ± 1.9* | 62 ± 3 | 6 |
|  |  | ***LF*** |  |  |
| **D280A** | 3.9 ± 1.2** | 22.9 ± 7.8** | 62 ± 6 | 5 |
| **T281A** | 2.4 ± 0.4* | 15.6 ± 2.3** | 72 ± 12 | 4 |
| **R282A** | 4.0 ± 0.3** | 14.7 ± 2.0** | 64 ±14 | 3 |
| **D283A** | 2.2 ± 0.6* | 17.1 ± 5.8** | 59 ± 10 | 6 |
| **L284A** | 0.8 ± 0.1 | 7.2 ± 1.0 | 60 ± 5 | 5 |
| **E285A** | 0.9 ± 0.2 | 8.5 ± 3.6 | 65 ± 2 | 3 |
| **H286A** | 4.0 ± 0.8** | 16.2 ± 1.6** | 55 ± 5 | 3 |
| **N287A** | 1.6 ± 0.1 | 10.2 ± 3.0 | 75 ± 16 | 3 |
| **V288A** | 1.7 ± 0.3 | 9.3 ± 1.7 | 72 ± 8 | 6 |
| **S289A** | 0.9 ± 0.3 | 11.3 ± 0.6 | 56 ± 7 | 6 |
| **P290A** | 0.8 ± 0.1 | 12.0 ± 1.5 | 53 ± 3 | 3 |
| **G291A** | 2.0 ± 0.5 | 16.5 ± 2.7** | 69 ± 12 | 5 |
| **Y292A** | 1.9 ± 0.2 | 15.8 ± 0.2** | 57 ± 3 | 10 |
| **N293A** | n.d. | n.d. | n.d. | n.d. |

## Desensitization time constants, τ_des1_ and τ_des2_, and their amplitudes, A_1_ and A_2_, were measured by biexponential fitting of current stimulated with 100 μM ATP for 60 s. The contribution of τ_des2_ was calculated in % as [A_2_ /(A_1+_A_2_)]; n= number of cells. Weighted desensitization time constant (τ_des_) is given in the last column of Table S1. The statistical significance was estimated by an ANOVA comparing the WT to the mutant receptors, p < 0.01 (**), p < 0.05 (*). Values could not be determined (n.d.).
